# Fc Gamma Receptors as Regulators of Bone Destruction in Inflammatory Arthritis

**DOI:** 10.3389/fimmu.2021.688201

**Published:** 2021-06-23

**Authors:** Yuyue Zuo, Guo-Min Deng

**Affiliations:** Department of Rheumatology and Immunology, Union Hospital, Tongji Medical College, Huazhong University of Science and Technology, Wuhan, China

**Keywords:** FcγRs, autoantibodies, osteoclasts, bone erosion, inflammatory arthritis

## Abstract

Bone erosion is one of the primary features of inflammatory arthritis and is caused by excessive differentiation and activation of osteoclasts. Fc gamma receptors (FcγRs) have been implicated in osteoclastogenesis. Our recent studies demonstrate that joint-deposited lupus IgG inhibited RANKL-induced osteoclastogenesis. FcγRI is required for RANKL-induced osteoclastogenesis and lupus IgG-induced signaling transduction. We reviewed the results of studies that analyzed the association between FcγRs and bone erosion in inflammatory arthritis. The analysis revealed the dual roles of FcγRs in bone destruction in inflammatory arthritis. Thus, IgG/FcγR signaling molecules may serve as potential therapeutic targets against bone erosion.

## Introduction

Inflammatory arthritis is a group of diseases characterized by joint inflammation and bone damage. About 0.1% of adults develop inflammatory arthritis annually (1). Rheumatoid arthritis (RA) is a chronic autoimmune disease characterized by progressive synovitis and bone destruction, causing irreversible joint damage and disability (1–4). Bone erosion is the central hallmark of RA in ultrasonography identification (5, 6). Anti-citrullinated protein antibodies (ACPAs) are considered to be among the leading risk factors for bone destruction in RA (7). Ankylosing spondylitis (AS) and psoriatic arthritis (PsA) are other common inflammatory arthritis diseases with bone destruction (8, 9).

Systemic lupus erythematosus (SLE) is a chronic autoimmune disease characterized by multi-organ tissue damage and high levels of autoantibodies in the serum (10). Arthritis is a common clinical manifestation with a prevalence of 69 to 95% in patients with SLE (11). However, only 4 to 6% of patients with SLE arthritis display bone erosion on plain radiographs (12–14). As to ACPA positive SLE patients, which are also called rhupus patients, they often overlapped clinical features and fulfilled American College of Rheumatology (ACR) criteria for RA classification (15, 16). It is still unclear why lupus arthritis without ACPA lacks bone destruction. Recently, FcγRs have been reported to exert a regulatory effect on osteoclastogenesis (17–25). Our recent study demonstrated that joint-deposited lupus IgG triggered arthritis without bone erosion in mice and lupus IgG inhibited osteoclastogenesis induced by receptor activator of nuclear factor kappa-B ligand (RANKL). FcγRI exerted an inhibitory effect of lupus IgG on RANKL-induced osteoclastogenesis (26). Our study suggests that FcγR could function as critical regulators of inflammatory arthritis.

Here, we review the published studies and demonstrate the association between the FcγR and bone erosion in inflammatory arthritis.

## Fcγ Receptor Family

**Figure 1:**
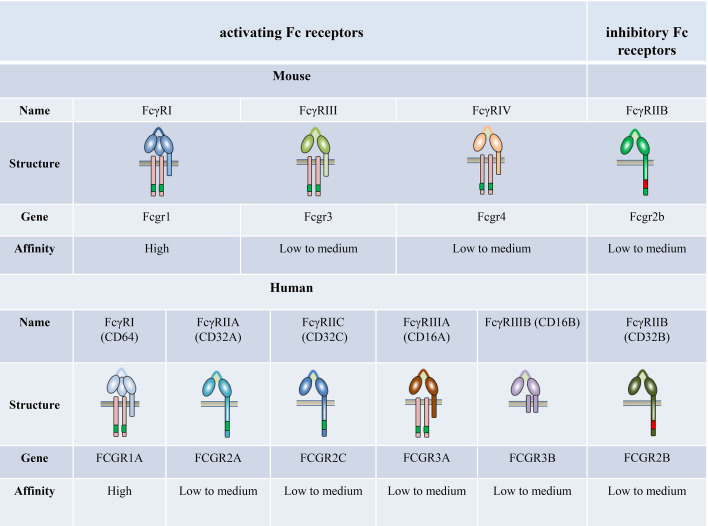
The family of classical Fc receptors for IgG. Schematic representations of FcγRs with respect to the cell membrane (brown bar), in complex with their respective signaling subunits. Mouse and humans have one high-affinity receptor, FcγRI; all other FcγRs have a low-to-medium affinity for the antibody Fc fragment.

FcγRs are receptors for the constant (Fc) region of IgG; these are expressed widely on the surface of immune cells, including monocytes, macrophages, neutrophils, dendritic cells (DCs), B cells, natural killer cells, and mast cells. Four different classes of FcγRs have been identified in mice, namely FcγRI, FcγRIIB, FcγRIII, and FcγRIV (27–29). The human and primate FcγR classifications are more complex. Humans possess six classic FcγRs with different IgG binding capacity and downstream signaling pathways: FcγRI (CD64), FcγRIIA (CD32A), FcγRIIB (CD32B), FcγRIIC (CD32C), FcγRIIIA (CD16A), and FcγRIIIB (CD16B), which are encoded by genes *FCGR1A*, *FCGR2A*, *FCGR2B*, *FCGR2C*, *FCGR3A*, and *FCGR3B*, respectively (**Figure 1**).

The affinity of FcγRs for IgG depends on the type of FcγR and IgG isotypes (30–36). FcγRI is the only known high-affinity FcγR (10^8^–10^9^ M^−1^) with a restricted isotype specificity. In contrast, FcγRII and FcγRIII have a low affinity for IgG (about 10^6^ M^−1^) with a broader isotype binding pattern (31, 32). FcγRIV is a novel receptor conserved across all mammalian species with an intermediate affinity (10^7^ M^−1^) and restricted subclass specificity (29, 37). FcγRIIIA is engaged by IgG1 and IgG2, whereas FcγRI and FcγRIV are engaged by IgG2 only (35). The affinity of mouse FcγRs is significantly higher compared with their corresponding human FcγRs (36).

FcγRs are divided into activating and inhibitory receptors and coexpressed on the same cell (38). Activating FcγRs, including FcγRI and FcγRIII, contain an immunoreceptor tyrosine-based activation (ITAM) in intracellular structure and transmit their signals *via* the ITAM, which recruits spleen tyrosine kinase (Syk) (39). FcγRIIB is the only known inhibitory FcγR with an immunoreceptor tyrosine-based inhibitory motif (ITIM) in its intracytoplasmic domain (40). The phosphorylation of ITIM counteracts the signals mediated by activating FcγRs (41–43). FcγRIIB is expressed widely on B cells, macrophages, and mast cells and downregulates several cellular functions, such as B-cell activation and mast cell degranulation (44). The activating-to-inhibitory (A/I) ratio on the same cell acts as the specific checkpoint for the arrest or progression of an immune response. Surprisingly, when monomeric or low-affinity immune complexes bind to activating FcγRs, the normally activating ITAM domain cannot induce co-aggregation of activating receptors, thereby partially phosphorylating the ITAM domain. Thus, partial tyrosine phosphorylation of ITAM by Src family kinases may result in the recruitment of inhibitory SHIP. This is called inhibitory ITAM (ITAMi) signal and is important in maintaining immune homeostasis (45–47).

Unlike other activating FcγRs, FcγRII proteins do not require the common FcR γ-chain for stable expression or function. They all have signaling motifs in their intracellular cytoplasmic domains (48). All the above FcγRs are transmembrane glycoproteins, except for human FcγRIIIB, which is expressed on neutrophils and is a glycophosphatidylinositol (GPI)-anchored protein (49, 50). The mechanisms by which FcγRIIIB transduces signals are still unknown (51).

## Fcγrs and Arthritis

During autoimmune diseases, such as RA and SLE, the autoantibodies and immune complexes cause inflammation *via* FcR aggregation (52). The altered expression of FcγRs on immune cells in the circulation and synovium of RA patients is the first indication of their involvement in inflammation (53–60). The absence of all FcγRs does not affect the number of osteoclast precursors or their osteoclastogenic potential. However, it reduces joint inflammation and bone erosion during inflammatory arthritis (61). FcγRIIB is particularly critical for maintaining the balance of an efficient inflammatory response or countering unwanted autoimmunity attacks. Multiple clinical studies have shown that FcγRIIB is a reliable biomarker for SLE susceptibility in different ethnic groups. FcγRIIB and its signaling pathways represent a vital checkpoint in peripheral and central tolerance and in controlling the development of autoreactive antibodies (62).

In addition to the altered expression of FcγRs, genetic variants associated with related single-nucleotide polymorphisms (SNPs) in populations with RA and lupus arthritis have been reported. Several genes encoding FcγRs that alter the affinity of FcγRs for IgGs have been described in several RA populations. In particular, some of these, such as the hFcγRIIa-R131 variant, which is related to an increased risk of developing RA, even influence the susceptibility to RA development and the response to treatment (63–70). In addition, an association between lupus arthritis and the FCGR2A as well as FCGR3A low copy number genotypes has been observed in Taiwan patients with SLE. The FCGR3A low copy number genotype was significantly enriched in patients with SLE having arthritis (71–73). Moreover, a meta-analysis revealed the association of the *FcγRIIa-R131* allele with SLE, especially in African Americans, whereas the *FcγRIIIa-F176* allele was associated with SLE in Caucasians and other groups (74). Furthermore, Tsang et al. demonstrated the association between low-affinity FcγR polymorphisms and susceptibility to SLE (75).

Studies using *FcγR* gene-deficient mice have greatly enhanced our understanding of the role of FcγRs in inflammatory arthritis (76, 77). The lack of activating FcγRs alleviates the disease severity in arthritis models (78–81). In different disease phases of inflammatory arthritis, the individual activating FcγRs have different significance (36, 61, 82–86). In the absence of FcγRI, FcγRIIB, and FcγRIIIA, FcγRIV is sufficient to induce arthritis alone (35). In contrast with activating FcγRs, the inhibitory FcγRIIB suppresses inflammation by inhibiting the activating signaling, as well as providing negative feedback on the production of autoantibodies by B cells (87–92).

Autoantibodies and their immune complexes play a central role in shaping a pro-inflammatory environment. Indeed, complexes of ACPA and rheumatoid factor (RF) induce the production of potent inflammatory cytokines (93–96). This effect is predominantly mediated by FcγR signaling on macrophages (51, 97). Tumor necrosis factor (TNF)-α, in combination with cytokines interleukin (IL)-4 and IL-13, downregulates FcγR-mediated function by decreasing the expression of activating FcγRs, suggesting that downregulated activating FcγRs might have an anti-inflammatory effect (98).

The Fc receptors on white blood cells are essential for effective phagocytosis of immune complexes and bacteria. Moreover, FcγRI is upregulated during infection. FcγRI (CD64) has previously been reported to distinguish systemic infections from inflammatory autoimmune diseases and viral infections. Patients without inflammatory and infectious conditions, such as osteoarthritis, have a lower level of neutrophil FcγRI than those with infections (99–104). Oppegaard et al. investigated the use of FcγRI in discerning septic arthritis from inflammatory joint disease and found that FcγRI is highly specific for infectious diseases, including septic arthritis. However, its sensitivity is poor in local infections (104). Although distinct meta-analyses have confirmed this, more large prospective studies need to be conducted to verify several cut-off values reported in the neutrophil FcγRI test in the clinical setting (105, 106).

Human and murine activating FcγRs are not functionally equivalent. A few studies performed in transgenic mice expressing human FcγRs examined their involvement in inflammatory arthritis (107). The results confirmed that the expression of the human FcγRIIA is associated with spontaneous autoimmune inflammation, with a crucial role in autoimmune diseases (92).

## FcγR Role in Bone Erosion

### Osteoclast Activation and Differentiation

**Figure 2:**
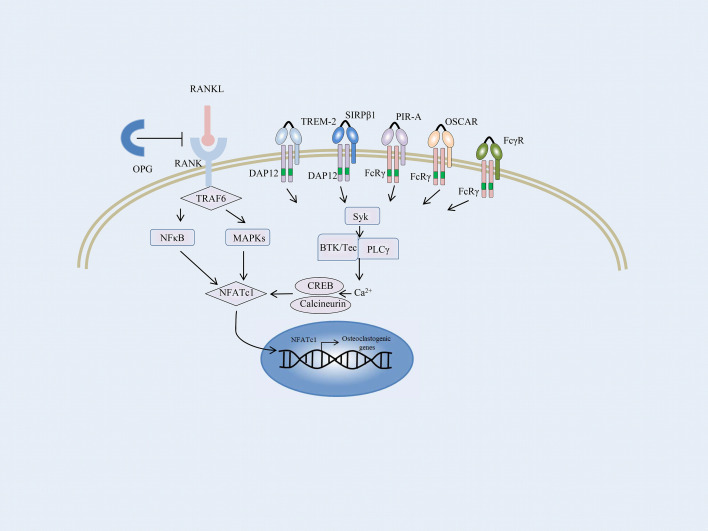
Overview of the osteoclast signaling network. A schematic representation of ITAM-mediated costimulatory signal in the RANKL-induced TRAF6 signaling pathway of osteoclast differentiation. In osteoclast precursors, phosphorylation of ITAM stimulated by immunoreceptors and RANKL–RANK interaction recruits the Syk family kinases, thus activating phospholipase Cγ (PLCγ), Bruton’s tyrosine kinase (BTK), as well as Tec kinases. They augment the calcium influx required for the activation of NFATc1. NFATc1 subsequently migrates to the nucleus, where it binds to its gene promoter and triggers an auto-amplifying feedback loop. Calcium signals in osteoclast precursors are provided by the ITAM-bearing proteins, Fc receptor γ subunit, and its functional analog DNAX activation protein of 12 kDa (DAP12). FcRγ-chain is associated with the immunoglobulin (Ig)-like receptors, such as osteoclast-associated receptor (OSCAR) and paired Ig-like receptor-A (PIR-A). DAP12 is associated with its signaling counterpart, triggering receptor expressed on myeloid cell-2 (TREM-2), and signal-regulatory protein β1 (SIRPβ1).

Bone balance depends on a dynamic regulation of bone formation and resorption, which are predominantly mediated by osteoblasts and osteoclasts, respectively (108, 109). Enhanced osteoclast activity could result in severe bone destruction as exemplified in autoimmune inflammatory diseases such as RA, whereas defective osteoblast differentiation causes diseases with a high bone mass, including osteopetrosis. Osteoclasts are the only bone-resorbing cells and play a central role in bone erosion. Osteoclasts are derived from multinucleated progenitors of the monocyte/macrophage family and are the link between immune and bone systems. RANK and RANKL are critical factors that together regulate osteoclast functions. In addition, macrophage colony-stimulating factor (M-CSF) is an essential cytokine in osteoclastogenesis (109–111). RANKL is majorly secreted by osteoblasts, osteocytes, T cells, and endothelial cells. And osteocytes express a much higher amount of RANKL required for osteoclastogenesis than osteoblasts (112, 113). The most important negative regulator of RANKL is the decoy receptor osteoprotegerin (OPG), which inhibits osteoclastogenesis by preventing RANKL–RANK interaction. The RANKL–RANK–OPG system modulates bone homeostasis by regulating osteoclasts (114). Osteoblasts and osteocytes also produce OPG to suppress osteoclastogenesis by masking RANKL signaling (115, 116). RANKL initiates osteoclastogenesis by inducing nuclear factor of activated T-cells, cytoplasmic 1 (NFATc1), *via* TNF receptor-associated factor 6 (TRAF6) and c-Fos pathways (117) (**Figure 2**). NFATc1 is the master transcription factor for pro-osteoclastogenic genes. In addition, several pro-inflammatory cytokines produced by innate immune cells and T cells, such as TNFα, IL-17, IL-1, and IL-6, stimulate osteoclastogenesis directly or indirectly (118).

### FcγRs and Osteoclastogenesis

Apart from the M-CSF and RANKL signaling, an ITAM costimulatory signal provided by the accessory protein for RANKL-RANK is required for osteoclastogenesis (119). Takayanagi et al. first reported that the activation of NFATc1 was insufficient for terminal differentiation of monocytes/macrophages into osteoclasts; calcium signals and calcineurin activation are essential for this process (117, 120). Calcium signals in myeloid cells are provided by the ITAM-bearing proteins, Fc receptor γ subunit, and its functional analog DNAX activation protein of 12 kDa (DAP12). Both the accessory proteins are intracellular adaptor molecules and play a crucial function in transducing the costimulatory signals for RANKL (121). Mice lacking the accessory proteins display a severe osteopetrotic phenotype with deficient osteoclast function (122).

FcRγ-chain is associated with immunoglobulin (Ig)-like receptors, such as osteoclast-associated receptor (OSCAR) and paired Ig-like receptor-A (PIR-A) (**Figure 2**). DAP12 is associated with its signaling counterpart, triggering receptor expressed on myeloid cell-2 (TREM-2), and signal-regulatory protein β1 (SIRPβ1), which are expressed on the cell membrane of osteoclast precursors and are essential for the communication between osteoclast precursors (33, 123). Activation of RANKL-RANK rapidly phosphorylates the ITAM motifs and recruits the protein kinase Syk, subsequently activating multiple downstream signaling cascades, such as phospholipase Cγ (PLCγ) and Bruton’s tyrosine kinase (BTK) as well as Tec kinases. They all enhance the effects of RANKL-signaling by augmenting the calcium influx required for the activation of NFATc1. NFATc1 subsequently migrates to the nucleus, where it binds to its gene promoter and triggers an auto-amplifying feedback loop (124, 125).

Osteoclasts and their precursors express FcγR (126), whereas FcγRI, FcγRIIB, and FcγRIIIA are significantly upregulated during human *ex vivo* osteoclastogenesis (127). Blocking of the FcR and deleting the FcγR gene reduce osteoclastogenesis stimulated by IgG complexes on osteoclast precursor cells (25). Although FcγRs are required for osteoclastogenesis and bone resorption in inflammatory disorders, their specific role in bone homeostasis is not completely understood.

### FcγRs and Bone Erosion

Costimulatory signals mediated by the ITAM motif cooperate with RANKL for bone homeostasis, suggesting the link between ITAM-harboring adaptors FcγRs and bone erosion (117). As an important link binding the bone system and immune system, FcγRs are not only receptors for the Fc portion of IgG but are also costimulatory molecules for RANKL-induced osteoclastogenesis (119, 121). Bone damage has been reported in seropositive RA patients before clinical disease onset, highlighting that osteoclastogenesis is independent of joint inflammation (128). This finding challenged the concept that inflammation is the primary trigger for bone erosion in inflammatory arthritis and indicated that bone loss might precede inflammation (129).

The expression of Fcγ receptors on osteoclast precursor cells and mature osteoclasts has been measured. FcγRI and FcγRIII are primarily expressed on human preosteoclasts, whereas the inhibitory FcγRIIB is majorly expressed on mature osteoclasts (127). Under physiological conditions, activating FcγRI and FcγRIV in mice does not have a major role in bone characteristics and osteoclast development (22). Bone homeostasis is not significantly different in mice with FcγRI or IV deficiency compared with wild mice (17). In addition, the deficiency of FcγRIIB does not affect osteoclastogenesis (23). Activating FcγRs transmit the positive signal. In contrast, FcγRIII functions as an inhibitory receptor in the differentiation of osteoclast precursor cells under physiological conditions. FcγRIII deprives the FcRγ subunit’s availability for other Ig-like receptors activating receptors, such as PIR-A and OSCAR, thus transmitting an ITAM-mediated inhibitory signal for osteoclastogenesis (130). Naive FcγRIII^–/–^mice have increased osteoclast numbers and an osteoporotic phenotype (22).

The relative importance of various FcγRs in osteoclastogenesis changes in the inflammatory arthritis microenvironment. Studies demonstrated that the stimulation of FcγRI and FcγRIV increases both osteoclast differentiation and function both *in vitro* and *in vivo* (22, 30). FcγRIII levels are increased, and FcγRIIB levels are decreased on bone marrow cells from mice with collagen-induced arthritis (CIA), indicating that FcγRIII induces osteoclastogenesis under inflammatory conditions (22). Furthermore, human RA patients with the *FcγRIIIa-158V* allele endure severe bone erosion compared with patients with the *FcγRIIIa-158F* allele (131, 132). Similarly, artificial crosslinking of FcγRI and FcγRIV leads to increased osteoclast differentiation without affecting their resorbing function *in vitro* (17). Osteoclast numbers and bone erosion were decreased in FcγRIV^–/–^mice compared with wild mice in a serum transfer model (17). FcγRIIB^–/–^mice spontaneously developed osteoporosis, which was reversed by an additional knockout of activating FcγRs (22).

De-sialylated IgGs binding to FcγRs with strong affinity have substantially high stimulatory effects on both murine and human osteoclasts (127, 133). In addition, IgGs were less sialylated during inflammation (22). Harre et al. confirmed that the interactions between immune complexes and osteoclasts were related to the degree of IgG sialylation, and only non-sialylated or low-sialylated immune complexes drive osteoclastogenesis. RA patients with low Fc sialylation levels of IgGs have significantly higher bone loss. The pro-osteoclastogenic effect of non-sialylated immune complexes is a common feature of all IgG antibodies (127). A recent study showed that in induced pluripotent stem cell derived mesenchymal stem cell (iMSCs), the sialylation degree of IgG determines the antibodies directed osteogenic potential by regulating immune responses and osteoclastogenesis (24),but desialylated IgG complexes do not affect arthritis-mediated bone loss (134).

Although the signaling of activating FcγRs mediated by immune complex increases osteoclast differentiation, different results exist for immune complex/FcγR on osteoclastogenesis and osteoclast function (**Table 1**). Previous studies demonstrated the immune complex-induced inhibition of osteoclastogenesis, which possibly acts *via* activating FcγRs (23, 139). This suggests that FcγRs may have dual roles in bone destruction in inflammatory arthritis. High levels of autoantibodies are a characteristic feature of SLE compared with other inflammatory arthritis (140, 141). The deposition of autoantibodies or immune complexes causes lupus nephritis (142), skin damage (143), splenomegaly (144), and damage to other organs. Lupus IgG can promote the differentiation of monocytes into DCs (145). These indicate that lupus autoantibodies may also play a protective role in bone destruction in inflammatory arthritis. Recently, our research results (26) demonstrated that joint-deposited lupus IgG induced arthritis without bone erosion by intraarticular injection of lupus IgG in mice. Monocytes/macrophages and their product TNFα are required for the development of lupus IgG-induced arthritis. To understand the mechanism of lupus IgG-induced arthritis with deficiency of bone erosion, we determined whether lupus IgG inhibited RANKL-induced osteoclastogenesis. We found that lupus IgG directly suppressed RANKL-induced osteoclastogenesis in a dose-dependent manner *in vitro*. The inhibitory effect of lupus IgG on osteoclastogenesis is related to timepoint in lupus IgG and RANKL treated macrophages. Deficiency of FcγRII and FcγRIII did not affect the inhibitory effect of lupus IgG on osteoclastogenesis, indicating that the inhibitory effect of lupus IgG on osteoclastogenesis is dependent on FcγRI. Lupus IgG and RANKL can downregulate the surface expression of FcγRI on bone marrow macrophages (20). Research results suggest that lupus IgG inhibits osteoclastogenesis by competitively occupying FcγRI on monocytes/macrophages and reducing RANKL signaling. The effect of activation or repression of RANKL-induced osteoclastogenesis depends on the extent of FcγRI occupancy by IgG. This protective mechanism explains non-destructive arthritis in SLE. In addition, it implies that FcγRI could be a therapeutic target for bone erosion in inflammatory arthritis.

**Table 1:** Different roles of FcγRs in arthritis and bone destruction.

FcγR subtype	Animal model	Function	Mechanism	Reference
FcγRI	CIA; K/BxN arthritis	activation	involving in the early arthritis pathology	(18)
	lupus-like arthritis;	activation OR inhibition	depending on the extent of FcγRI occupancy by IgG and RANKL	(26)
FcγRIIA	CIA; K/BxN arthritis	activation	crosstalking with C5a receptor; driving the osteoclastogenesis independent of RANKL and inflammatory cytokines by binding to IgG-ICs	(107, 135)
FcγRIIB	AIA; CIA; lupus-like disease in FcγRIIB−/− mice	inhibition	inhibition of FcγRI/III; efficient clearance and endocytosis of ICs	(18, 89, 136, 137)
FcγRIII	CIA; K/BxN arthritis	activation	being required for early arthritis onset	(18, 35)
FcγRIV	AIA; K/BxN arthritis	activation	cross-linking with ICs directly; inducing the influx of S100A8/A9-producing neutrophils into the arthritic joint.	(5, 17, 35)
Unclassified	K/BxN arthritis	inhibition	activating FcγRs, but not FcγRIIB mediate IC-induced inhibition of osteoclastogenesis	(23)
	CIA; IC-induced bone destruction	activation OR inhibition	the relative expression of FcγRI/III/IV and FcγRIIB; the availability of ICs	(22)
	CIA	activation OR inhibition	the degree of IgG sialylation determines the effect of FcγRs	(127)
	TNF-induced osteolysis model	inhibition	cross-linking of FcγRs with IVIG suppresses osteoclastogenesis by inducing A20 expression.	(138)

CIA, collagen-induced arthritis; AIA, antigen-induced arthritis; IC, immune complex; IgG, immunoglobulin G; IVIG, Intravenous immunoglobulin; RANKL, receptor activator of nuclear factor kappa-B ligand; TNF, tumor necrosis factor.

The deposition of ACPA is important for osteoclastogenesis in RA (146). Different studies have identified that ACPA prevalence is significantly increased in SLE patients with erosive arthritis (16). Recent studies have explored the direct effect of ACPA-mediated bone erosion. ACPA IgG together with their citrullinated antigens forms immune complexes that stimulate immune cells *via* their interaction with FcγRs (93, 147). By using polyclonal ACPAs purified from ACPA-containing serum of RA patients, Harre et al. provided the first validation that ACPAs can directly promote osteoclast differentiation and activation (7). ACPA IgG might affect osteoclastogenesis by the activation of Fc receptors on osteoclasts directly. IgG Fc sialylation is crucial for immune complex–osteoclast interactions (127). Besides, ACPA IgG is shown less sialylated than random IgG (148). There are other published papers regarding the detailed mechanisms of ACPA’s direct regulation, but the exact mechanism of ACPA’s direct effect on erosion remains unclear (149–152).

## FcγR Immunotherapy

The crucial role of FcγRs in both inflammatory arthritis and bone erosion may offer a promising therapeutic target for bone destruction in inflammatory arthritis. One indirect mechanism involves the neutralization of autoimmune IgG Fc by soluble FcγRs, These drugs include the recombinant soluble FcγIIB receptor SM101 (NCT03851341) and monoclonal antibody targeted the receptors. For example, antagonistic monoclonal antibody against the hFcγRIIIA has been shown to be effective in a patient with immune thrombocytopenia (ITP) refractory to all conventional therapies (153). And human recombinant soluble FcγRIIB treatment could ameliorate collagen-induced arthritis by reducing immune complexes-stimulated inflammation and joint swelling (154). Besides, recombinant human soluble FcγRII was evaluated as an effective therapeutic strategy in inhibiting chronic murine lupus pathology (155).

Another mechanism involves the direct blocking of the IgG-binding site on FcγRs. Recombinant multimeric Fc fragments with a high affinity for FcγRs have been shown to be efficacious in animal models of RA, ITP, and graft-versus-host disease (GVHD) (156). These include PF-06755347 (NCT03275740), CSL730 (NCT04446000) and CSL777 (Preclinical) (157). However, nonspecific crosslinking of activating FcγRs could lead to undesired clinical adverse events, and monovalent antibody derivatives, such as Fab, may reduce severe clinical adverse events (158). Up to now, results of above molecules from clinical trials have been promising in autoimmune diseases, but further long-term data are needed (159, 160).

Intravenous immunoglobulin (IVIG) treatment is efficient in several different immune disorders (161, 162). IVIG consists predominantly of IgG and a small fraction of immune complexes. It exerts anti-inflammatory effects in both humans and animal models by its Fc but not Fab fragments (163). Besides, previous studies confirmed that IVIG could directly inhibits human osteoclastogenesis by suppressing the RANK signaling, the suppressive effect is partly mediated by IgG immune complexes contained within IVIG preparations (138). Our study showed that lupus IgG induced synovial inflammation but inhibited RANKL-induced osteoclastogenesis. The suppressive effect is mediated by the competitive occupation of FcγRI on monocytes/macrophages (26).

## Conclusions and Future Perspectives

Bone erosions are remarkable features in inflammatory arthritis, such as RA, but not in lupus arthritis. Osteoclasts are major cells for bone erosions. Activating FcγR containing ITAM motifs is required for RANKL-induced osteoclastogenesis. FcγR can effectively regulate inflammatory arthritis and bone erosions. Based on published studies, we conclude that FcγR may have dual roles in osteoclastogenesis. The effect of activating and inhibiting osteoclastogenesis depends on the extent of FcγRI occupancy by IgG and RANKL, respectively. Specific IgG molecules or Fc fragments with a high affinity for FcγRI designed to occupy FcγRI may exert the inhibitory effect on bone erosion. The sialylation level of IgG Fc binding to FcγRs needs to be taken into account as well. A deeper understanding of FcγRs involved in physiological and pathological osteoclastogenesis will be valuable in identifying new targets and developing potential therapeutic strategies for inflammatory arthritis.

## Data Availability Statement

The raw data supporting the conclusions of this article will be made available by the authors, without undue reservation.

## Author Contributions

YZ and G-MD designed the manuscript and figures. YZ and G-MD drafted the manuscript and approved the final version of the manuscript. G-MD revised the final version of the manuscript critically. All authors contributed to the article and approved the submitted version.

## Funding

This study was supported by the initiating fund of Union Hospital, Tongji Medical College, Huazhong University of Science and Technology (G-MD, 02.03.2018-41).

## Conflict of Interest

The authors declare that the research was conducted in the absence of any commercial or financial relationships that could be construed as a potential conflict of interest.

The reviewer PZ declared a shared affiliation with the authors to the handling editor at the time of review.
